# The age-related effect on cognitive performance in cognitively healthy elderly is mainly caused by underlying AD pathology or cerebrovascular lesions: implications for cutoffs regarding cognitive impairment

**DOI:** 10.1186/s13195-020-00592-8

**Published:** 2020-03-24

**Authors:** Emma Borland, Erik Stomrud, Danielle van Westen, Oskar Hansson, Sebastian Palmqvist

**Affiliations:** 1 Clinical Memory Research Unit, Department of Clinical Sciences, Lund University, Malmö, Sweden; 2 Department of Neurology, Skåne University Hospital, Malmö, Sweden; 3 Memory Clinic, Skåne University Hospital, Malmö, Sweden; 4 Department of Neuroradiology, Lund University, Lund, Sweden

**Keywords:** Cognitive assessment, True norms, Robust norms, Cutoff, Normative, Age, Preclinical pathology

## Abstract

**Background:**

As research in treatments for neurocognitive diseases progresses, there is an increasing need to identify cognitive decline in the earliest stages of disease for initiation of treatment in addition to determining the efficacy of treatment. For early identification, accurate cognitive tests cutoff values for cognitive impairment are essential.

**Methods:**

We conducted a study on 297 cognitively healthy elderly people from the BioFINDER study and created subgroups excluding people with signs of underlying neuropathology, i.e., abnormal cerebrospinal fluid [CSF] β-amyloid or phosphorylated tau, CSF neurofilament light (neurodegeneration), or cerebrovascular pathology. We compared cognitive test results between groups and examined the age effect on cognitive test results.

**Results:**

In our subcohort without any measurable pathology (*n* = 120), participants achieved better test scores and significantly stricter cutoffs for cognitive impairment for almost all the examined tests. The age effect in this subcohort disappeared for all cognitive tests, apart from some attention/executive tests, predominantly explained by the exclusion of cerebrovascular pathology.

**Conclusion:**

Our study illustrates a new approach to establish normative data that could be useful to identify earlier cognitive changes in preclinical dementias. Future studies need to investigate if there is a genuine effect of healthy aging on cognitive tests or if this age effect is a proxy for higher prevalence of preclinical neurodegenerative diseases.

**Suppementary informationl:**

**Supplementary information** accompanies this paper at 10.1186/s13195-020-00592-8.

## Background

Cognitive test norms are used for comparing a person’s performance to a large group of individuals of the same age, education, and gender. Norms based on persons without underlying cerebral pathology are crucial for identification of cognitive decline in neurocognitive disorders at early stages. To accurately capture the earliest declines in progression, it is necessary to know at what cognitive performance a cognitive decline should be suspected. It is preferable to capture an individual’s actual changes in cognitive level; however, when there are no available longitudinal data, reference data from cognitively healthy individuals can be used for comparison. A mild decline in cognitive function can be caused by underlying pathology, such as beta-amyloid [1, 2], neurofibrillary tangles, cerebral infarctions, and Lewy bodies [3], and it is well known that pathophysiologic processes of neurocognitive disorders begin many years before a diagnosis of dementia [4, 5]. Research on neurocognitive disorders, such as Alzheimer disease (AD), has therefore switched focus from diagnosing clinically symptomatic AD, to a biological definition of the disease, in order to identify preclinical individuals with only subtle cognitive decline for earlier recognition and intervention [5, 6].

Traditionally, test norms were established from subjectively cognitively healthy people, which later was improved by creating robust test norms using longitudinal data from people without clinical progression in cognitive symptoms [7–11]. Further, cognitive test scores have been stratified by age, as test results are correlated with age in cognitively healthy persons [12]. These previous methods did not account for the presence of relevant cerebral pathologies and might thus have a lower sensitivity for identifying subtle cognitive changes that accompany preclinical dementias.

In this study, we hypothesized that (1) excluding cognitively healthy controls with underlying measurable in vivo brain pathologies, would result in improved test results and more strict cutoffs for cognitive impairment, and (2) the effect of age on cognitive test results to a large extent is driven by individuals with preclinical neurodegenerative disease, and by excluding these from normative studies, the effect of age would be reduced. To examine this, we compared test results from cognitively healthy people based on the earlier methods explained above, with test results from people without certain brain pathologies, including abnormal accumulation of beta-amyloid (Aβ) and elevated phosphorylated tau (P-tau) as a marker for Alzheimer pathology (measured using cerebrospinal fluid [CSF] Aβ42/40 and P-tau), cerebrovascular lesions (measured using magnetic resonance imaging [MRI]), or axonal injury (measured using CSF neurofilament light (NfL)). We established cognitive test norms and cutoffs for cognitive impairment from cognitively healthy elderly persons without these underlying pathologies and compared these to cutoffs in traditionally classified cognitively healthy controls. We also investigated the effect of age, years of education, and gender on cognitive tests in groups with and without underlying measurable in vivo pathologies.

## Methods

### Participants

Cognitively healthy elderly persons were included from the prospective Swedish BioFINDER Study (http://biofinder.se), and participants for this study were enrolled between July 6, 2009, to March 4, 2015. The population was consecutively included from the large population-based Malmö Diet and Cancer Study [13]. The inclusion criteria for being included as a healthy control in BioFINDER were (1) a score on MMSE ≥ 28 points at the screening visit, (2) age ≥ 60 years old, and (3) fluent in the Swedish language. The exclusion criteria were (1) presence of subjective cognitive impairment, (2) presence of significant neurological disease (e.g., stroke, Parkinson disease, multiple sclerosis), (3) significant unstable systemic illness or organ failure that makes it difficult to participate in the study, (4) severe psychiatric disease (e.g., severe depression or psychotic syndrome), (5) dementia or mild cognitive impairment (MCI), and (6) current significant alcohol or substance misuse. Eligible participants were evaluated by physicians well-experienced in dementia disorders regarding cognitive status, fulfillment of inclusion criteria and absence of exclusion criteria in a 1-h-long interview including a semi-structured interview for Clinical Dementia Rating (CDR) [14] scoring. Only subjects with a complete data set of CDR at baseline and at least one follow-up visit (either a 2- or 4-year follow-up), available CSF ratio of Aβ42/40, CSF P-tau, CSF NfL, assessment of white matter lesions (WML), and cortical infarctions were included. This resulted in a study population of 297 participants. In a subanalysis, we also applied different test cutoffs on both the controls and patients with subjective cognitive decline (SCD), collectively termed cognitively unimpaired [5] (*n* = 529) from BioFINDER. The study criteria for the SCD cohort have been described elsewhere [15].

### Procedures

#### Cognitive tests

Eight cognitive tests were examined in the present study, covering the cognitive domains of executive function, attention, episodic, and semantic memory as well as visuospatial function. The Alzheimer’s Disease Assessment Scale (ADAS) is an instrument designed specifically to evaluate the severity of cognitive and noncognitive behavioral dysfunctions characteristic of people with AD. In this study, we used ADAS Naming Objects and Fingers (here ADAS naming), where the subject is assessed in their ability to name different objects and fingers, and ADAS 10-word delayed recall (here ADAS-delayed recall), where the participant was previously exposed to 10 words during three learning trials, and memory function is tested for delayed recall. For both ADAS subscales, points are counted as words the subject does not acknowledge or recall, meaning higher points equals worse test results [16]. Animal Fluency is a semantic verbal fluency test where the participant is asked to produce as many words in the category “animals” as possible in 60 s. This tests for cognitive flexibility when shifting between animal categories (e.g., horse–cow–sheep and dog–cat–mouse) related to frontal functioning, and clustering related to temporal lobe disturbance [17]. In this study, we scored the number of animals the individual produces. A Quick Test of Cognitive Speed – Color and Form (AQT) consists of 40 figures with different colors and forms, assessing cognitive processing speed and task-switching. The time it takes to name the color and form for all figures equals the test score [18]. Stroop color and Word Test (here Stroop) assesses the ability to inhibit the cognitive interference that occurs when the processing of one stimulus affects the simultaneous processing of another stimulus [19]. The participant reads words printed in another color, leading to a prefrontal cortex activation [20]. The time it takes to complete the test becomes the test score. Trail Making Test A and B (TMT A and B) provide information on visual search, speed of processing, mental flexibility, and executive functions [21]. In TMT A, the participant draws lines between 25 numbers sequentially, and in TMT B participants alternate between sequential numbers and letters (i.e., 1–A, 2–B, 3–C etc.). The time it takes to complete the test equals the test score [21]. For Symbol Digit Modalities Test (SDMT), the participant uses a reference key to pair specific symbols with numbers, receiving one point for every correct answer within the response time of 90 s. The test assesses divided attention, visual scanning, tracking, and motor speed [20].

#### MRI

All participants were examined using the same 3-T MRI scanner [22]. White matter lesions and cortical infarctions were assessed by visual inspection for all the participants. White matter lesions were graded using the 4-point Fazekas scale (0–3) on T2 FLAIR images, and participants with a Fazekas score ≥ 2 were defined as having clinically significant cerebrovascular pathology [23]. Presence of supra- or infracortical infarctions were assessed on 2D FLAIR and T1-weighted MPPRAGE images. Liquidated tissue with or without surrounding gliosis was regarded as infarction and included if ≥ 2 mm in size.

#### CDR assessment

Eligible participants were thoroughly assessed at the Memory Clinic, Skåne University Hospital, at baseline and at least a 2- or 4-year follow-up by a physician well experienced in dementia disorders. This assessment included a semi-structured CDR interview. CDR is a numeric scale scoring 0–3 points used to quantify the severity of symptoms of dementia based on measures of memory, orientation, judgment and problem solving, community affairs, home and hobbies and personal care [14].

#### CSF analysis

The procedure and analysis of CSF followed the Alzheimer’s Association Flow chart for CSF biomarkers [15, 24]. CSF Aβ42, Aβ40, and P-tau were analyzed using Elecsys immunoassays on all participants. CSF NfL was analyzed with ELISAs (NF-light® ELISA kit; UmanDiagnostics AB, Umeå, Sweden) as previously described [25].

#### Creating different normative samples

The participants in our study were all included in the sample cohort (A) of 297 people (see under “Participants”). Four different subgroups (B, C, D, and E) were created based on different exclusion criteria. In cohort B, we excluded persons with clinically progressive cognitive decline according to CDR during ≥ 2 years. For all participants, CDR assessments were conducted at baseline and at a 2-year follow-up except for two participants with missing data at 2-year follow-up for whom data from the 4-year visit was used; these two participants had not progressed in CDR. Clinical progression was defined as a CDR sum of boxes ≥ 0.5, corresponding to the traditional method of establishing robust test norms [7–10]. In cohort C, we excluded persons with any AD pathology, here defined as either abnormal Aβ or tau. Abnormality was defined using previously established cut-offs. For Aβ, it was a CSF Aβ42/40 ratio of < 0.059 [26] and for tau a CSF P-tau level ≥ 28 pg/mL [27]. In cohort D, we excluded persons with cerebrovascular pathology (white-matter lesions or infarctions), defined by a Fazekas score ≥ 2 in any brain region and/or any visual cortical infarctions on MRI scans. In cohort E, we excluded people with any underlying measurable in vivo pathology; abnormal CSF Aβ or tau pathology, cerebrovascular pathology or CSF NfL, a marker of neurodegeneration that is affected also in frontotemporal dementia [28] and atypical Parkinson diseases [29] or simply a measure of neurodegeneration [5, 30]. The CSF NfL cutoff for cohort E was created with mixture modeling statistics [31] on cognitively healthy controls and patients with subjective cognitive decline or mild cognitive impairment in BioFINDER (*n* = 823). CSF NfL-levels were logarithmized because of skew distribution and several outliers, and the cutoff was defined as log NfL > 3.33 pg/mL.

#### Statistical analysis

Mean scores and standard deviations were established for tests of executive function (TMT A and B, SDMT, Stroop, and Animal Fluency), attention (AQT), episodic memory (ADAS-delayed recall), and semantic memory (ADAS naming). Correlation coefficients for age and test results were calculated using Spearman correlation. To investigate if the absence of significant correlations for most cognitive tests was caused by a lack of statistical power, we conducted bootstrap analysis with 500 bootstraps from 100 individuals (to ensure bootstrapping of the smallest cohort (cohort E) would not result in finding significance when gaining a larger cohort and more statistical power) and calculated mean correlation values as well as mean *p* values. Association between years of education and test results were conducted with Spearman correlation and association between gender and test results with Mann-Whitney. The association between groups and cognitive test results controlled for age, education and sex were calculated using multivariable linear regression with test score as outcome and disease pathology as a predictor (0 = present or 1 = absent). Cutoff values for cognitive impairment for the cognitive tests were calculated by adding/subtracting 1.5 standard deviations (SD) to/from the mean values, e.g., for SDMT where the aim is to achieve as many points as possible, 1.5 SD is subtracted from the mean score to calculate the cutoff value, whereas for TMT, 1.5 SD was added to mean scores. Two-sided *p* value of < 0.05 indicated statistical significance. The above analyses were all calculated with SPSS Statistics version 25. Confidence intervals for the cutoffs and correlation coefficients were estimated from 500 bootstrap samples using R Version 3.5.2.

## Results

### Demographics

The total study population (cohort A) consisted of 297 persons between the ages of 64–88 years old, mean age 73.5 years (see Table 1 for demographics).

**Table 1:** Demographics

	A. Study cohort	B. No progress in CDR	C. No amyloid or tau pathology	D. No vascular pathology	E. No measurable in vivo pathology
Participants, *N*	297	278	223	161	120
MMSE score, mean (SD)^a^	29.1 (0.9)	29.1 (0.9)	29.1 (0.9)	29.1 (0.9)	29.1 (0.9)
Age, mean (SD)	73.5 (5.0)	73.4 (5.0)	73.4 (5.0)	72.5 (4.6)	72.2 (4.6)
Education years, mean (SD)	12.3 (3.7)	12.3 (3.8)	12.2 (3.6)	12.5 (0.9)	12.6 (3.5)
Gender					
Male	39.4%	38.8%	39.5%	41.6%	42.5%
Female	60.6%	61.2%	60.5%	58.4%	57.5%
APOE ε4 (≥ 1 allele)	27.3%	25.9%	16.3%	30.8%	20.3%
Prevalence of abnormal biomarkers					
CSF Aβ42/40 < 0.059	24.9%	23.0%	0%	24.2%	0%
CSF P-tau > 28 pg/mL	15.2%	13.3%	0%	14.3%	0%
Log CSF NfL > 3.33 pg/mL	2.4%	1.8%	2.2%	1.2%	0%
White matter lesions^b^	45.5%	43.9%	44.8%	0%	0%
≥ 1 Cortical infarctions	1.3%	1.1%	1.7%	0%	0%
Comorbidity					
Hypertension	43.4%	42.8%	43.5%	41.0%	43.3%
Diabetes	10.1%	10.4%	9.4%	11.2%	10.0%
Ischemic heart disease	6.7%	6.8%	6.3%	4.3%	2.5%

^a^All groups have a high MMSE score due to the inclusion criteria of MMSE ≥ 28 points. ^b^Measured as Fazekas score ≥ 2 point in any region

### Correlation between age and cognitive test results

In the total population (cohort A) and the cohort without amyloid or tau pathology (cohort C), there was a significant association between age and all other cognitive test results. In cohort B (no clinical progression in CDR score), there was no significant association between age and ADAS-delayed recall or ADAS naming. In the cohort excluding only cerebrovascular pathology (cohort D), there was no significant correlation between age and test results for ADAS-delayed recall, ADAS naming, Animal Fluency, or AQT, but there was for Stroop, TMT A and B, and SDMT (i.e., a remaining age effect for tests of attention and executive function). In the cohort without any measurable pathology (cohort E), the age effect only remained for TMT A and TMT B (Table 2). The age effect remained for difference scores (TMT-B minus TMT-A) for all cohorts (Additional table 1). To examine if the absence of significant correlations for most cognitive tests was caused by a lack of statistical power (*n* = 297 in cohort A, *n* = 120 in cohort E), we performed further sensitivity analyses. We created 500 bootstrap samples from cohorts A and E, respectively, with 100 participants in each sample. The mean *p* values for Spearman correlations in the 500 bootstrap samples from cohort A (with only *n* = 100) still indicated significant correlations between age and all cognitive tests as in the original analysis. For cohort E, there were only significant correlations for age and TMT A and B in the bootstrap analysis, indicating that the difference in age effect on cognitive test results between cohort A and E was not caused by a difference in statistical power (Additional table 2).

**Table 2:**
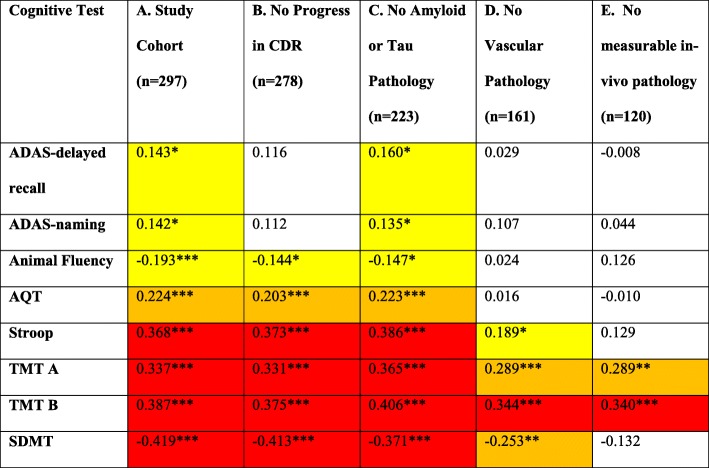
Correlation coefficients for test results and age in each cohort

Correlation coefficients for age and test results conducted with Spearman correlation. Only significant correlation coefficients are colored. Yellow boxes for coefficients ≥0.1 to <0.2, orange boxes for ≥0.2 to <0.3, red boxes for ≥0.3. *Correlation is significant at the 0.05 level, **correlation is significant at the 0.01 level ***correlation is significant at the 0.001 level

### The association between cognitive test results, gender, and education

Education correlated with test results for all tests apart from ADAS-delayed recall, ADAS naming, and TMT A in cohort A, where higher education was associated with improved test scores. In cohort E, there was an education effect for ADAS-delayed recall, Stroop, TMT B, and SDMT (Additional table 3). We found a significant association between female gender and ADAS-delayed recall in cohorts A, B, and C, and between male gender and improved test scores in AQT for cohort D. No significant differences were found between men and women for any other cognitive tests regardless of cohort (A–E) (Additional table 4).

### Establishing cutoffs for cognitive impairment

**Fig. 1:**
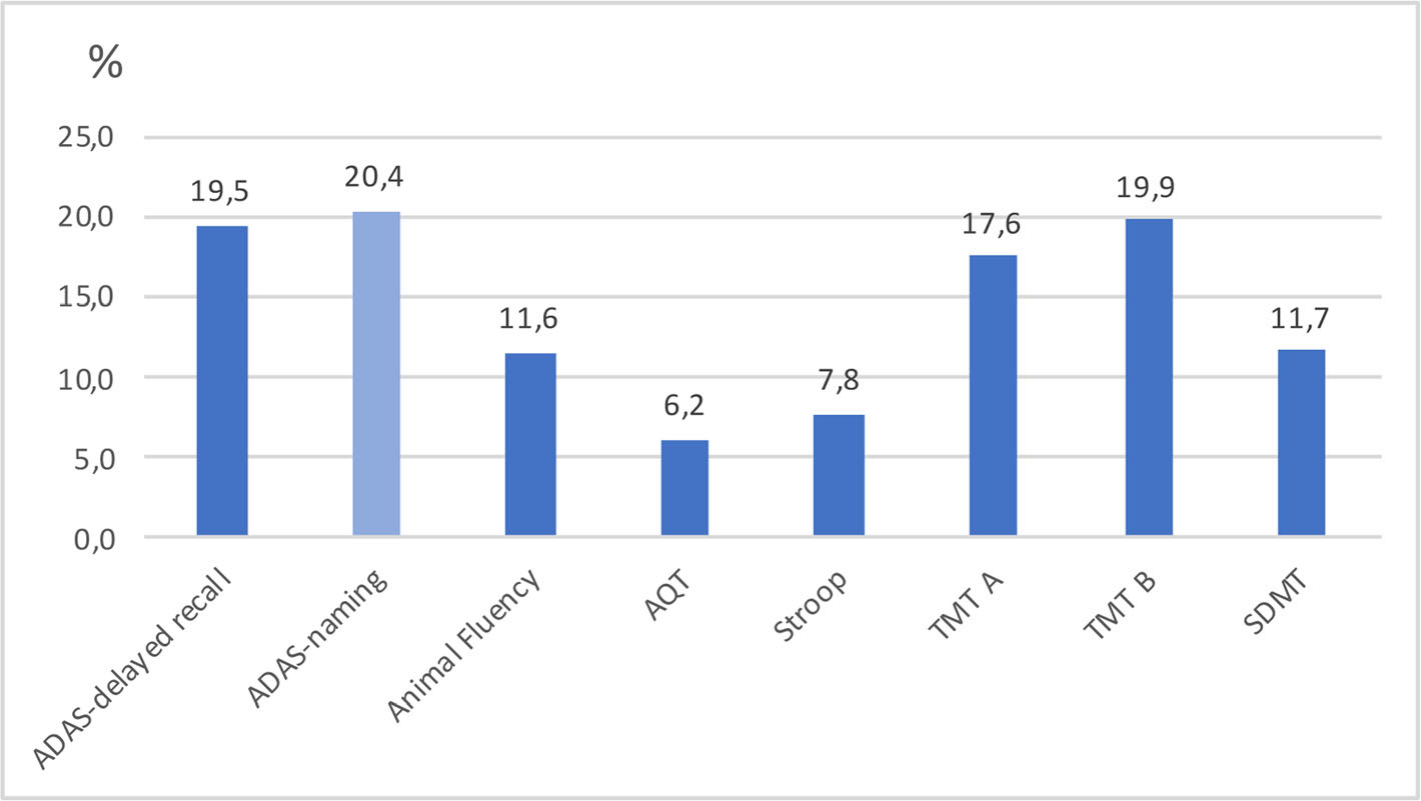
Percent change in test cutoffs (1.5 SD from mean) between the total population and those without measurable brain pathologies. All test cutoffs were significantly stricter in cohort E (no measurable pathologies) compared to cohort A (whole population) except for ADAS naming (Table 4). Percent changes were calculated by dividing cutoffs for cohort E with cutoffs for cohort A

In Table 3, we present mean test scores of each cognitive test for all five cohorts. The cutoff for cognitive impairment was defined as the mean value ± 1.5 SD (depending on whether higher score equaled worse or better performance) [32, 33]. In cohort E, all cutoffs for cognitive impairment differed significantly (were stricter) from the total population (cohort A) apart from ADAS naming. Cutoffs in cohort E differed significantly from the traditional method for establishing robust norms (cohort B) for Animal Fluency, TMT A, TMT B, and SDMT (Table 4). In Fig. 1, we present percent differences in cutoffs (at ± 1.5 SD from mean) between the total cohort (cohort A) and the cohort without pathology (cohort E). Of those cutoffs that differed significantly, the cutoff was 6.2 to 19.9% stricter in cohort E. All test results showed an improvement in cognitive test score when excluding those with underlying pathologies. The effect of the underlying pathology on the cognitive test result was independent of age, gender, and education (Additional table 5). We also investigated the sensitivity for detecting preclinical pathology using the cutoffs from cohort E (without underlying pathology) compared to cutoffs from cohort A (the total population) in cognitively unimpaired individuals in BioFINDER (controls and patients with subjective cognitive decline, *N* = 528). Overall, we found that using cutoffs from group E increased the sensitivity for detecting preclinical AD or cerebrovascular pathology and also improved the overall performance (higher Youden index), compared to using cutoffs from group A (Additional tables 6–7).

**Table 3:** Cognitive test results for each cohort

Cognitive test	A. Study cohort	B. No progress in CDR	C. No amyloid or tau pathology	D. No vascular pathology	E. No measurable in vivo pathology
ADAS-delayed recall	1.98 (1.93)	1.79 (1.72)	1.80 (1.78)	1.81 (1.80)	1.59 (1.56)
ADAS naming	0.38 (0.79)	0.34 (0.74)	0.36 (0.79)	0.30 (0.75)	0.24 (0.68)
Animal Fluency	21.7 (5.5)	22.1 (5.4)	22.0 (5.5)	22.4 (5.3)	23.0 (5.3)
AQT	66.0 (12.9)	65.1 (12.3)	65.8 (12.9)	63.8 (12.0)	63.3 (11.1)
Stroop	28.9 (7.4)	28.5 (7.0)	28.6 (7.5)	27.5 (6.9)	26.9 (6.7)
TMT A	46.0 (17.0)	45.6 (17.1)	45.6 (16.9)	43.0 (14.8)	41.0 (12.0)
TMT B	104.4 (50.8)	101.8 (49.4)	101.9 (49.0)	97.1 (44.7)	90.0 (36.5)
SDMT	37.0 (8.4)	37.5 (8.3)	37.5 (8.5)	38.4 (8.3)	39.1 (8.0)

Data are shown as mean (SD). A: The entire population. B: No progress in Clinical Dementia Rating (CDR) over 2 years. C: No preclinical AD (i.e., CSF Aβ42/40 and P-tau not abnormal). D: No vascular pathology. E: No measurable pathology (i.e., no AD pathology, cerebrovascular pathology or increased NfL)

**Table 4:** Cognitive test cutoffs at 1.5 SD from mean for cohorts A, B and E

	Cohort A cutoffs (95% CI)	Cohort B cutoffs (95% CI)	Cohort E cutoffs (95% CI)
ADAS-delayed recall	**4.88 (4.59–5.16)**	4.38 (4.13–4.61)	3.93 (3.63–4.20)
ADAS naming	1.57 (1.42–1.71)	1.44 (1.29–1.58)	1.25 (1.01–1.48)
Animal Fluency	**13.5 (13.1–13.9)**	**14.0 (13.6–14.4)**	15.0 (14.4–15.7)
AQT	**85.3 (83.6–86.9)**	83.5 (81.8–85.2)	80.0 (77.8–82.1)
Stroop	**40.0 (39.0–40.9)**	39.0 (38.0–39.8)	36.9 (35.1–38.6)
TMT A	**71.5 (69.2–73.9)**	**71.3 (68.9–73.8)**	59.0 (56.5–61.2)
TMT B	**180.6 (172.1–189.0)**	**176.0 (167.8–184.4)**	144.6 (135.5–152.5)
SDMT	**24.3 (23.7–25.0)**	**25.1 (24.4–25.8)**	27.2 (26.1–28.3)

Cutoffs (1.5 SD from mean) created from 500 bootstrap samples. We found significantly improved cutoffs in cohort E compared to cohort A for all tests apart from ADAS naming. We found significantly improved cutoffs in cohort E compared to the traditional method of creating robust norms (cohort B) for Animal Fluency, TMT A, TMT B, and SDMT, i.e., non-overlapping 95% CIs (presented in bold)

## Discussion

In this study, we examined cognitive test scores, cutoffs for cognitive impairment, and age effects in 297 cognitively healthy elderly people with and without certain brain pathologies, including cerebrovascular pathology on MRI, CSF Aβ42/40, and P-tau as markers for preclinical AD and CSF NfL as a marker of neurodegeneration. The significant, age-related effect on cognitive test results disappeared for cognitive tests when excluding people with underlying preclinical pathology (the effect was predominantly caused by the exclusion of cerebrovascular pathology) for all tests, except for TMT A and TMT B that test attention/executive function (Table 2). This somewhat fits with a previous paper showing that age-related decline to a large extent can be explained by underlying brain markers such as cortical thickness, different regional brain volumes, structural connectivity, and white matter hyperintensities [34], suggesting age itself does not affect cognition but is a confounder for the association between underlying brain markers or disease mechanisms and cognitive test results.

When excluding the cognitively healthy elderly with underlying pathologies, we found significantly stricter cutoff values for cognitive impairment for all the examined cognitive tests, apart from ADAS naming, compared to the total cohort of cognitively healthy elderly (Fig. 1, Table 4). The improvement in cognitive test score when excluding underlying pathologies was independent of age, gender, and education (Additional table 5). To our knowledge, this is the first study that illustrates the effect of producing test norms from certified cognitively normal individuals without underlying AD pathology or cerebrovascular pathology.

In the total population (cohort A) and the cohort without amyloid or tau pathology (cohort C), we found a significant correlation between age and all test results, which is congruent with the common notion that aging is accompanied by cognitive decline [35] and that most cognitive test results are affected by age [2, 36], regardless of level of amyloid [37]. There are however previous studies showing more prominent effects of preclinical AD (i.e., AD pathology in cognitively healthy controls) on cognition [2, 38, 39]. There could be several causes for why we do not find similar results in our study. Firstly, when examining the effect of underlying pathologies on test results (Additional table 5) on AD-negative participants, we are only comparing with a small number of participants with AD pathology (*N* = 74), i.e., there could be a lack of statistical power. Secondly, we use CSF measures of amyloid pathology instead of amyloid-PET as in other previous studies, which is more closely related to cognition [38, 40]. Differences in results could also be cutoff dependent. Nonetheless, we also want to point out that (supporting the present findings) there are several large studies showing that the effect of AD pathology is not always apparent at baseline, but instead becomes apparent after longitudinal follow-ups of around 4 years (as shown in, e.g., the BioFINDER, AiBL, and ADNI studies [41, 42]).

For cohort B, there were no age effects for ADAS delayed recall or ADAS naming. When excluding people with cerebrovascular pathology (cohort D), the age effect on cognitive test scores disappeared for ADAS-delayed recall, ADAS naming, Animal Fluency, and AQT, besides, was reduced for the remaining tests. In our cohort without measurable pathology (E), we found similar results for age effect on cognitive tests as in cohort D, though the age effect disappears even for Stroop and SDMT. However, the above suggests vascular pathology stands for most of the age effect.

For the cognitive tests TMT A and TMT B, a significant age correlation remained even when excluding people with underlying pathologies. This suggests either that there is an actual cognitive decline related to a seemingly healthy aging process or that the age-related decline is caused by an unmeasurable pathology or cause. The latter case is a limitation of this study and also of the current research field in general. Unaccounted pathologies that could cause cognitive decline, such as TDP-43 [43] in, e.g., Limbic-predominant age-related TDP-43 encephalopathy (LATE) [44] or frontotemporal dementias and alpha-synuclein in parkinsonian disorders, are not yet possible to measure in vivo and could therefore not be excluded from our study. Other possible causes of the remaining age-related decline in test results which are not accounted for are difficulties in psychomotor speed, gait speed, or decline in visual competence. However, these abilities are included in other cognitive tests where the age effect disappeared, speaking against them being the cause of the remaining age effect. Speed variables are frequently found to have moderate to large relations with age across adulthood; however there are many types of measures of speed in cognitive testing [45]. Different types of speed are tested in TMT A (motor speed) and B (motor speed and internal responses) [46], SDMT (processing speed as well as motor speed), Stroop (processing speed), and Animal Fluency (mental speed) [20], suggesting decrease in motor speed is the main cause of remaining age-related decrease in TMT A and B. To test the age effect on internal responses in our study, we calculated the difference score (TMT-B minus TMT-A) for each cohort. Mean difference scores in cohort A was 57.9 s and in cohort E 48.4 s, suggesting time for internal responses decreases in cohort E. Analyses, however, showed that the age effect for the difference score remained for all cohorts (see Additional table 1). Previous studies have however suggested the relationship between TMT A and B is more complicated than only differing in motor speed, as TMT B has a longer distance and visual scanning is more difficult [47]. In another perspective, it has previously been suggested that the degree of slowing in speed is greater for tasks involving spatial information than for those involving verbal information [48], which could suit with our findings that the age effect still remains for TMT A and B but disappears for others testing speed such as Animal Fluency, AQT, and Stroop, all including verbal information.

Our findings that cutoffs change when excluding patients with preclinical pathology confirms the findings from the two previous studies that excluded underlying AD pathology [2, 39]. Cutoffs for cohort E were also stricter than the traditional method for establishing robust norms (cohort B) in Animal Fluency, TMT A, TMT B, and SDMT (Table 4). We also found reduced SD for all tests in cohort E compared to cohorts A and B (Table 3), implicating the variance in test scores was smaller for cohort E and, thus, that it was a more homogeneous group than the total population. We do not, however, believe that the present study was less stringent in clinically ruling out cognitive impairment, since previously published normative scores are more in line with those from group A (total population) than the ones from cohort E (without underlying pathology) [17, 20, 49, 50].

To our knowledge, no other study has excluded cognitively healthy participants based on biomarkers of vascular, amyloid, P-tau, and neurodegenerative abnormalities to investigate the effect on test norms and the relationship with age. However, Hassenstab et. al. investigated test norms on patients without positive biomarkers for preclinical AD (T-tau, P-tau, and Aβ42/40), as well as abnormal hippocampal volume [39]. As in our study, they assessed patients with TMT A, TMT B, and Animal Fluency. They found slightly improved cutoffs (mean + 1.5 SD) for TMT A at 46.7 s and 118.7 s for TMT B for a younger (< 75 years) group, however slightly worse for persons ≥ 75 years of age (TMT A of 52.2 s and TMT B 148.8 s) closer to cohort E’s cutoffs of TMT A 59.0 s and 144.6 s for TMT B. For Animal Fluency however, they found less strict cutoffs for both younger and older subjects, 13.4 vs. 11.2 words, compared to our cutoff at 15.0 words. In their study, variance in cognitive performance attributable to age remained for TMT A, TMT B, and Animal Fluency in their group without preclinical AD, however, was less profound than in the preclinical group. Another previous study investigated test results on persons without positive biomarkers for Aβ for people aged > 40 years. Means of cognitive tests including TMT A and B were calculated. Cutoffs (mean + 1.5 SD) for Aβ-negative individuals were 51.3 s for TMT A and 123.8 s for TMT B, lower than cohort E’s cutoffs of TMT A 59.0 s and 144.6 s for TMT B, but could be explained by their young cohort [2].

In terms of implementing stricter cutoffs such as those from group E, there is a potential risk of overdiagnosing cognitive impairment in a clinical setting. They are therefore probably not always suitable in, e.g., a primary care setting. However, in order to screen for preclinical disease such as in the enrolment process of clinical AD trials or a tentative future scenario where disease-modifying AD treatments are available, such new and more sensitive cutoffs could be suitable to use as a first screening step before applying biomarkers to verify any potential underlying pathology. This potential is hinted at in our analysis when using the cutoffs for detecting preclinical AD and cerebrovascular lesions in the cognitive unimpaired participants in BioFINDER (Additional tables 6–7). But as our cutoffs are produced from a small cohort of only 120 participants, we do not suggest they are the definite cutoffs to be used for this purpose (nor that these tests necessarily are the most suitable). Instead, we believe this study illustrates the change in cutoff values when removing underlying pathologies and, most importantly, the findings problematize the concept of normal cognitive aging and the previously used method of stratifying cutoffs according to age*.* For future normative studies, we suggest that in addition to excluding individuals with subjective cognitive impairment, dementia, MCI, alcohol or substance misuse, or any other condition prone to cause cognitive impairment as usually is the case for control cohorts, we also propose that one considers excluding subjects with underlying cerebrovascular pathology, AD pathology, or signs of neurodegeneration. To simplify the establishment of such test norms from large population-based materials, it may be possible to use plasma biomarkers instead of invasive lumbar punctures or PET scans to screen for AD pathology (e.g., plasma P-tau that performs similar to CSF P-tau [51] or the combination of plasma P-tau181 and plasma Aβ42/40 [52, 53]) and axonal/cerebrovascular lesions (e.g., plasma NfL [54, 55]).

An advantage with this study is our consecutively included cohort of individuals assessed with CSF biomarkers, structural neuroimaging, and cognitive assessments including longitudinal CDR. A weakness of our study is the relatively small group without any pathology (120 people). However, we showed that the difference between the total study cohort (A) and those without any pathology (E) regarding the age effect on cognition was not caused by a difference in statistical power (Additional table 2). Further, we chose to not include MRI measures of atrophy, partly because of the uncertainty of which brain regions to measure, and partly because brain atrophy is a downstream marker, i.e., possibly caused by old age and not just neurocognitive diseases. Instead, we chose to investigate CSF NfL as a measure of axonal damage and neurodegeneration, as it can identify preclinical diseases for which we have no other specific diagnostic biomarker, such as atypical Parkinson disease and diseases in the frontotemporal lobe disease spectrum as well as act as a general marker for neurodegeneration [5, 28–30].

## Conclusions

In summary, we propose that most age-related decline in cognitive tests is caused by underlying pathological mechanisms and that the traditionally used age stratification might have been caused by a higher proportion of preclinical neurocognitive disorders in the older age groups. Stratifying norms according to age could result in a delayed identification of early cognitive decline in early stages of neurocognitive diseases, especially due to cerebrovascular pathology. Further research with a larger population is necessary to confirm our findings that the age effect disappears for most cognitive tests in people without underlying pathology.

## Supplementary information

Additional file 1: **Table S1.** Correlation coefficients for difference scores in Trail Making Test measures with age in each cohort.

Additional file 2: Table S2. Mean correlation coefficients and *p*-values between test results and age from bootstrap analysis.

Additional file 3: Table S3. Correlation between years of education and test results.

Additional file 4: Table S4. Associations between gender and cognitive test scores.

Additional file 5: Table S5. Linear regression models examining the effect of underlying pathologies on test results.

Additional file 6: Table S6. Comparison between cutoffs from cohort A and E for detecting preclinical AD in cognitively unimpaired BioFINDER participants.

Additional file 7: Table S7. Comparison between cutoffs from cohort A and E for detecting preclinical cerebrovascular disease in cognitively unimpaired BioFINDER participants.

Additional file 8: Table S8. Cutoffs (+/− 1.5 SD from mean) for all cognitive tests in different groups.
