# Supplementary material for: The age-related effect on cognitive performance in cognitively healthy elderly is mainly caused by underlying AD pathology or cerebrovascular lesions: implications for cutoffs regarding cognitive impairment
Source: Alzheimers Res Ther. 2020 Mar 24;12:30. doi: 10.1186/s13195-020-00592-8 (PMC7093968; doi:10.1186/s13195-020-00592-8)
Supplement: Supplementary file 1 — Table S1. Correlation coefficients for difference scores in Trail Making Test measures with age in each cohort. [file 13195_2020_592_MOESM1_ESM.docx]

**Additional table 1. Correlation coefficients for difference scores in Trail Making Test measures with age in each cohort**

| **Cognitive Test** | **A. Study Cohort** | **B. No Progress in CDR** | **C. No Amyloid or Tau Pathology** | **D. No Vascular Pathology** | **E. No measurable in-vivo pathology** |
| --- | --- | --- | --- | --- | --- |
| **TMT B – TMT A** | 0.302*** | 0.283*** | 0.305*** | 0.297*** | 0.260** |

*Correlation coefficients for age and test results conducted with Spearman correlation. Only significant correlation coefficients are colored. Yellow boxes for coefficients ≥0.1 to <0.2, orange boxes for ≥0.2 to <0.3, red boxes for ≥0.3. *Correlation is significant at the 0.05 level, **correlation is significant at the 0.01 level, ***correlation is significant at the 0.001 level.*
