# Supplementary material for: The age-related effect on cognitive performance in cognitively healthy elderly is mainly caused by underlying AD pathology or cerebrovascular lesions: implications for cutoffs regarding cognitive impairment
Source: Alzheimers Res Ther. 2020 Mar 24;12:30. doi: 10.1186/s13195-020-00592-8 (PMC7093968; doi:10.1186/s13195-020-00592-8)
Supplement: Supplementary file 2 — Mean correlation coefficients and p-values between test results and age from bootstrap analysis. [file 13195_2020_592_MOESM2_ESM.docx]

**Additional table 2. Mean correlation coefficients and p-values between test results and age from bootstrap analysis**

| **Cognitive test** | **A. Study Cohort (n=100)** | **B. No Progress in CDR (n=100)** | **C. No Amyloid or Tau Pathology (n=100)** | **D. No Vascular Pathology (n=100)** | **E. No measurable in-vivo pathology (n=100)** |
| --- | --- | --- | --- | --- | --- |
| **ADAS-delayed recall** | 0.142* | 0.115* | 0.162* | 0.029 | -0.009 |
| **ADAS-naming** | 0.142* | 0.114* | 0.136* | 0.107 | 0.045 |
| **Animal Fluency** | -0.193*** | -0.144* | -0.148* | 0.023 | 0.126 |
| **AQT** | 0.224*** | 0.202*** | 0.224*** | 0.018 | -0.011 |
| **Stroop** | 0.368*** | 0.374*** | 0.386*** | 0.187* | 0.130 |
| **TMT A** | 0.337*** | 0.331*** | 0.365*** | 0.290*** | 0.289** |
| **TMT B** | 0.387*** | 0.376*** | 0.405*** | 0.344*** | 0.340*** |
| **SDMT** | -0.420*** | -0.413*** | -0.371*** | -0.253** | -0.134 |

*Calculated mean correlation values and mean p-value conducted with bootstrap analysis with 500 bootstraps from 100 individuals. *Correlation is significant at the 0.05 level, **correlation is significant at the 0.01 level, ***correlation is significant at the 0.001 level. Yellow boxes for coefficients ≥0.1 to <0.2, orange boxes for ≥0.2 to <0.3, red boxes for ≥0.3.*
