# Supplementary material for: The age-related effect on cognitive performance in cognitively healthy elderly is mainly caused by underlying AD pathology or cerebrovascular lesions: implications for cutoffs regarding cognitive impairment
Source: Alzheimers Res Ther. 2020 Mar 24;12:30. doi: 10.1186/s13195-020-00592-8 (PMC7093968; doi:10.1186/s13195-020-00592-8)
Supplement: Supplementary file 3 — Correlation between years of education and test results. [file 13195_2020_592_MOESM3_ESM.docx]

**Additional table 3. Correlation between years of education and test results**

| **Cognitive test** | **A. Study Cohort (n=297)** | **B. No Progress in CDR (n=278)** | **C. No Amyloid or Tau Pathology (n=223)** | **D. No Vascular Pathology (n=161)** | **E. No measurable in-vivo pathology (n=120)** |
| --- | --- | --- | --- | --- | --- |
| **ADAS-delayed recall** | -0.094 | -0.097 | -0.094 | -0.187* | -0.224* |
| **ADAS-naming** | -0.102 | -0.129* | -0.094 | -0.148 | -0.113 |
| **Animal Fluency** | 0.183** | 0.200*** | 0.172** | 0.124 | 0.093 |
| **AQT** | -0.132* | -0.153* | -0.130 | -0.107 | -0.132 |
| **Stroop** | -0.148* | -0.157** | -0.196** | -0.195* | -0.256** |
| **TMT A** | -0.107 | -0.103 | -0.121 | -0.153 | -0.169 |
| **TMT B** | -0.168** | -0.170** | -0.196** | -0.210* | -0.279** |
| **SDMT** | 0.270*** | 0.268*** | 0.281*** | 0.276*** | 0.258** |

*Correlation coefficients conducted with Spearman test between test result and education for each group. Only significant correlation coefficients are colored. Yellow boxes for coefficients ≥0.1 and <0.2, orange boxes for ≥0.2 and <0.3. *Correlation is significant at the 0.05 level, **correlation is significant at the 0.01 level ***correlation is significant at the 0.001 level.*
