# Supplementary material for: The age-related effect on cognitive performance in cognitively healthy elderly is mainly caused by underlying AD pathology or cerebrovascular lesions: implications for cutoffs regarding cognitive impairment
Source: Alzheimers Res Ther. 2020 Mar 24;12:30. doi: 10.1186/s13195-020-00592-8 (PMC7093968; doi:10.1186/s13195-020-00592-8)
Supplement: Supplementary file 4 — Associations between gender and cognitive test scores. [file 13195_2020_592_MOESM4_ESM.docx]

**Additional table 4. Associations between gender and cognitive test scores**

| **Mean test results** | **A. Study Cohort** | | **B. No Progress in CDR** | | **C. No Amyloid or Tau Pathology** | | **D. No Vascular Pathology** | | **E. No measurable in-vivo pathology** | |
| --- | --- | --- | --- | --- | --- | --- | --- | --- | --- | --- |
| **Gender** | **M** | **F** | **M** | **F** | **M** | **F** | **M** | **F** | **M** | **F** |
| **ADAS-delayed recall** | **2.35 (1.84)**  **(n=117)** | **1.74*****  **(1.96)**  **(n=180)** | **2.18**  **(1.71)**  **(n=108)** | **1.55*****  **(1.69)**  **(n=170)** | **2.05 (1.67)**  **(n=88)** | **1.64***  **(1.83)**  **(n=135)** | 2.01 (1.74)  (n=67) | 1.67 (1.83)  (n=94) | 1.75 (1.62)  (n=51) | 1.48 (1.51)  (n=69) |
| **AQT** | 65.6 (12.8)  (n=117) | 66.2 (13.0)  (n=179) | 64.3 (11.4)  (n=108) | 65.5 (12.8)  (n=169) | 65.6 (13.0)  (n=88) | 66.0  (13.0)  (n=134) | **61.9* (11.8)**  **(n=67)** | **65.2 (12.1)**  **(n=93)** | 61.7 (11.1)  (n=51) | 64.5 (11.1)  (n=68) |

*Data are shown as mean (SD). Table showing a significant difference (data in bold) in test results between gender for ADAS-delayed recall in cohort A-C, where women have better test results, and for AQT in cohort D, where men have significantly better test results. No significant differences were found between gender and other cognitive tests regardless of cohort (A-E). P-values calculated with Mann-Whitney test with test results as dependent variable and gender as independent groups. *Correlation is significant at the 0.05 level, ** correlation is significant at the 0.01 level, ***correlation is significant at the 0.001 level. Acronyms: F, female; M, male.*
