# Supplementary material for: The age-related effect on cognitive performance in cognitively healthy elderly is mainly caused by underlying AD pathology or cerebrovascular lesions: implications for cutoffs regarding cognitive impairment
Source: Alzheimers Res Ther. 2020 Mar 24;12:30. doi: 10.1186/s13195-020-00592-8 (PMC7093968; doi:10.1186/s13195-020-00592-8)
Supplement: Supplementary file 5 — Linear regression models examining the effect of underlying pathologies on test results. [file 13195_2020_592_MOESM5_ESM.docx]

**Additional table 5. Linear regression models examining the effect of underlying pathologies on test results**

|  | **C. No Amyloid or Tau Pathology** | **D. No Vascular Pathology** | **E. No measurable in-vivo pathology** |
| --- | --- | --- | --- |
| **ADAS-delayed recall** | **-0.708**  **0.005**** | -0.242  0.284 | **-0.551**  **0.016*** |
| **ADAS-naming** | -0.058  0.582 | -0.127  0.180 | **-0.188**  **0.049*** |
| **Animal Fluency** | 1.079  0.134 | 1.042  0.105 | **1.645**  **0.011*** |
| **AQT** | -0.134  0.937 | **-3.358**  **0.025*** | **-3.195**  **0.036*** |
| **Stroop** | -0.790  0.399 | **-1.966**  **0.018*** | **-2.395**  **0.004**** |
| **TMT A** | -0.865  0.686 | **-3.803**  **0.045*** | **-5.822**  **0.002**** |
| **TMT B** | -6.977  0.308 | -7.030  0.242 | **-15.908**  **0.009**** |
| **SDMT** | 1.804  0.074 | 1.296  0.149 | **1.986**  **0.028*** |

*Linear regression using test score as outcome and disease pathology as a predictor (0=present or 1=absent; i.e. not a comparison with subgroup C/D/E vs group A). The table shows unstandardized estimates for disease pathology, adjusted for age, gender and education. P-values <0.05 are enhanced in bold. *Correlation is significant at the 0.05 level, ** correlation is significant at the 0.01 level, *** correlation is significant at the 0.001 level.*
