# Supplementary material for: The age-related effect on cognitive performance in cognitively healthy elderly is mainly caused by underlying AD pathology or cerebrovascular lesions: implications for cutoffs regarding cognitive impairment
Source: Alzheimers Res Ther. 2020 Mar 24;12:30. doi: 10.1186/s13195-020-00592-8 (PMC7093968; doi:10.1186/s13195-020-00592-8)
Supplement: Supplementary file 6 — Comparison between cutoffs from cohort A and E for detecting preclinical AD in cognitively unimpaired BioFINDER participants. [file 13195_2020_592_MOESM6_ESM.docx]

**Additional table 6. Comparison between cutoffs from cohort A and E for detecting preclinical AD in cognitively unimpaired BioFINDER participants**

|  |  | **ADAS-delayed recall** | **ADAS-naming*** | **Animal fluency** | **AQT** | **Stroop** | **TMT A** | **TMT B** | **SDMT** |
| --- | --- | --- | --- | --- | --- | --- | --- | --- | --- |
| **Cutoffs from Group A** | **Sensitivity:** | 30.72% | 11.84% | 16.00% | 13.73% | 11.85% | 13.33% | 5.11% | 9.77% |
|  | **Specificity:** | 86.33% | 91.67% | 93.01% | 87.94% | 90.54% | 93.08% | 94.87% | 92.74% |
|  | **Youden index:** | 0.17 | 0.04 | 0.09 | 0.02 | 0.02 | 0.06 | -0.00 | 0.03 |
| **Cutoffs from Group E** | **Sensitivity:** | 43.14% | 11.84% | 21.33% | 18.95% | 20.00% | 22.96% | 13.14% | 19.55% |
|  | **Specificity:** | 77.21% | 91.67% | 90.05% | 82.57% | 83.60% | 84.28% | 90.60% | 87.70% |
|  | **Youden index:** | 0.20 | 0.04 | 0.11 | 0.02 | 0.04 | 0.07 | 0.04 | 0.07 |

*Comparison between preclinical AD (N=153; positive CSF Aβ42/40 and P-tau or only positive CSF Aβ42/40 according to cutoffs described in methods) vs. all others (N =375). *No significant difference in cutoff scores between groups (1.25 for cohort E vs 1.57 for cohort A).*
