# Supplementary material for: The age-related effect on cognitive performance in cognitively healthy elderly is mainly caused by underlying AD pathology or cerebrovascular lesions: implications for cutoffs regarding cognitive impairment
Source: Alzheimers Res Ther. 2020 Mar 24;12:30. doi: 10.1186/s13195-020-00592-8 (PMC7093968; doi:10.1186/s13195-020-00592-8)
Supplement: Supplementary file 7 — Comparison between cutoffs from cohort A and E for detecting preclinical cerebrovascular disease in cognitively unimpaired BioFINDER participants. [file 13195_2020_592_MOESM7_ESM.docx]

**Additional table 7. Comparison between cutoffs from cohort A and E for detecting preclinical cerebrovascular disease in cognitively unimpaired BioFINDER participants**

|  |  | **ADAS-delayed recall** | **ADAS-naming*** | **Animal fluency** | **AQT** | **Stroop** | **TMT A** | **TMT B** | **SDMT** |
| --- | --- | --- | --- | --- | --- | --- | --- | --- | --- |
| **Cutoffs from Group A** | **Sensitivity:** | 24.91% | 11.36% | 13.36% | 15.38% | 13.16% | 11.84% | 5.74% | 10.67% |
|  | **Specificity:** | 87.04% | 92.28% | 93.95% | 89.84% | 92.86% | 94.31% | 95.22% | 94.79% |
|  | **Youden index:** | 0.12 | 0.04 | 0.07 | 0.05 | 0.06 | 0.06 | 0.01 | 0.05 |
| **Cutoffs from Group E** | **Sensitivity:** | 35.47% | 11.36% | 17.18% | 21.54% | 22.81% | 23.68% | 13.11% | 18.22% |
|  | **Specificity:** | 78.14% | 92.28% | 90.73% | 85.77% | 87.62% | 87.68% | 91.74% | 89.57% |
|  | **Youden index:** | 0.14 | 0.04 | 0.08 | 0.07 | 0.10 | 0.11 | 0.05 | 0.08 |

*Comparison between preclinical cerebrovascular disease (N =269; any Fazekas score >1 or cortical infarctions according to cutoffs described in methods) vs. all others (N =251*)*. *No significant difference in cutoff scores between groups (1.25 for cohort E vs 1.57 for cohort A).*
