# Supplementary material for: The age-related effect on cognitive performance in cognitively healthy elderly is mainly caused by underlying AD pathology or cerebrovascular lesions: implications for cutoffs regarding cognitive impairment
Source: Alzheimers Res Ther. 2020 Mar 24;12:30. doi: 10.1186/s13195-020-00592-8 (PMC7093968; doi:10.1186/s13195-020-00592-8)
Supplement: Supplementary file 8 — Cutoffs (+/− 1.5 SD from mean) for all cognitive tests in different groups. [file 13195_2020_592_MOESM8_ESM.docx]

**Additional table 8. Cutoffs** **(+/-1.5 SD from mean) for all cognitive tests in different groups**

|  | **Cohort A cutoffs**  **(95% CI)** | **Cohort B cutoffs (95% CI)** | **Cohort C cutoffs (95% CI)** | **Cohort D cutoffs (95% CI)** | **Cohort E cutoffs**  **(95% CI)** |
| --- | --- | --- | --- | --- | --- |
| **ADAS-delayed recall** | 4.88 (4.59 – 5.16) | 4.38 (4.13 – 4.61) | 4.47 (4.16 – 4.75) | 4.51 (4.16 – 4.85) | 3.93 (3.63 – 4.20) |
| **ADAS-naming** | 1.57 (1.42 – 1.71) | 1.44 (1.29 – 1.58) | 1.55 (1.36 – 1.73) | 1.43 (1.22 – 1.63) | 1.25 (1.01 – 1.48) |
| **Animal fluency** | 13.5 (13.1 – 13.9) | 14.0 (13.6 – 14.4) | 13.8 (13.3 – 14.3) | 14.5 (14.1 – 15.1) | 15.0 (14.4 – 15.7) |
| **AQT** | 85.3 (83.6 – 86.9) | 83.5 (81.8 – 85.2) | 85.3 (83.5 – 87.1) | 81.9 (79.6 – 83.9) | 80.0 (77.8 – 82.1) |
| **Stroop** | 40.0 (39.0 – 40.9) | 39.0 (38.0 – 39.8) | 39.8 (38.7 – 41.0) | 37.9 (36.5 – 39.2) | 36.9 (35.1 – 38.6) |
| **TMT A** | 71.5 (69.2 – 73.9) | 71.3 (68.9 – 73.8) | 70.9 (68.2 – 73.7) | 65.2 (62.4 – 67.8) | 59.0 (56.5 – 61.2) |
| **TMT B** | 180.6 (172.1 – 189.0) | 176.0 (167.8 – 184.4) | 175.5 (167.0 – 184.3) | 164.2 (154.2 – 173.2) | 144.6 (135.5 – 152.5) |
| **SDMT** | 24.3 (23.7 – 25.0) | 25.1 (24.4 – 25.8) | 24.8 (24.0 – 25.7) | 25.9 (25.0 – 26.8) | 27.2 (26.1 – 28.3) |

*Cutoffs (1.5 SD from mean) created from 500 bootstrap samples.*
